# Predictive value of serum TBA for 2-year MACEs in ACS patients undergoing PCI: a prospective cohort study

**DOI:** 10.1038/s41598-023-50304-z

**Published:** 2024-01-19

**Authors:** Wen Wen, Qinze Li, Jianqing She, Xiaofang Bai, Lisha Zhang, Ruifeng Li, Yan Wu, Juan Zhou, Zuyi Yuan

**Affiliations:** 1 Department of Ultrasound, Clinical Medical College, First Affiliated Hospital of Chengdu Medical College, Chengdu, China; 2 Department of Cardiovascular Medicine, First Affiliated Hospital, Xi’an Jiaotong University, Xi’an, China; 3 Department of Ultrasound, First Affiliated Hospital, Xi’an Jiaotong University, Xi’an, China; 4 Department of Cardiology, Second Affiliated Hospital of Xi’an Jiaotong University, Xi’an, China; 5 Key Laboratory of Molecular Cardiology, Xi’an, Shaanxi Province China; 6 Key Laboratory of Environment and Genes Related to Diseases (Xi’an Jiaotong University), Ministry of Education, Xi’an, China

**Keywords:** Biomarkers, Cardiology, Diseases, Endocrinology, Risk factors

## Abstract

Bile acids play important roles in lipid metabolism and glucose homeostasis. Limited research exist on the association between serum total bile acid (TBA) levels and major adverse cardiovascular events (MACEs) in patients with acute coronary syndrome (ACS), particularly those with comorbid type 2 diabetes mellitus (T2DM). This study was conducted to examine the relationship between baseline serum TBA level and T2DM status in patients with ACS after percutaneous coronary intervention (PCI) and to identify the predictive value of TBA levels for a 2-year risk of MACEs. 425 ACS patients underwent PCI were recruited and divided into three groups based on baseline serum TBA concentration. An analysis of the association between the T2DM status and baseline serum TBA levels was conducted using univariate linear regression and multivariate linear regression. The predictive relevance of serum TBA levels was evaluated using the receiver operating characteristic (ROC) curve and Cox regression. Kaplan–Meier curves were employed to analyze the differences among groups in predicting MACEs over a 2-year follow-up period. Baseline serum TBA levels were higher in ACS patients who were diagnosed with T2DM (the median 3.6 µmol/L) than those without T2DM (the median 3.0 µmol/L). T2DM status in ACS patients was positively correlated with baseline serum TBA concentrations (β: 1.7, 95% confidence interval [CI] 0.3–3.0), particularly in the male (β: 2.0, 95% CI 0.3–3.6) and 50–69-year-old (β: 2.5, 95% CI 0.6–4.4) populations. The areas under the ROC curve of baseline serum TBA levels predicted MACEs in ACS and ACS-T2DM patients following PCI were 0.649 (95% CI 0.595–0.703) and 0.783 (95% CI 0.685–0.881), respectively. Furthermore, Cox regression analysis showed that baseline serum TBA level was associated with the occurrence of MACEs in patients with ACS after PCI over a 2-year follow-up period, especially in those diagnosed with T2DM, whose baseline TBA concentration was lower than 10.0 µmol/L. ACS Patients with T2DM had higher serum TBA levels. TBA level at baseline was an independent predictor of MACEs in ACS patients who underwent PCI, especially with comorbid T2DM.

## Introduction

Bile acids (BA), converted by excessive cholesterol, which can be excreted from feces as bile salts, are known to play a vital role in cholesterol homeostasis^1,2^. Abnormal BA metabolism can lead to cholesterol metabolic disturbances, resulting in the deposition of excessive low-density lipoprotein cholesterol (LDL-C) in arterial intima, which is believed to be the reason for the progression of atherosclerosis (AS). It is reasonable to speculate that circulating BA may increase the risk of coronary artery disease (CAD)^3^; however, the exact relationship between circulating BA and CAD is still controversial. Diabetic patients are more likely to suffer from CAD than those without it^4^. Although revascularization and antithrombotic strategies have achieved quite a success in decreasing the mortality of patients with acute coronary syndrome (ACS), the outcomes are unsatisfactory, especially in individuals with type 2 diabetes mellitus (T2DM)^5^. Several studies have reported that BA metabolic disturbances may result in an imbalance of glucose and energy metabolism, preceding the occurrence of T2DM^6,7^. Increased serum total bile acids (TBA) levels may lead to T2DM progress, supporting the potential role of BA metabolism in T2DM pathogenesis^8,9^.

A previous study^10^ carried out in our laboratory revealed that circulating TBA levels were highly related to the severity of CAD in patients. We hypothesize that TBA can be a fast and convenient candidate marker for predicting the risk of major adverse cardiovascular events (MACEs) in ACS patients following percutaneous coronary intervention (PCI) over a long period of time. The current study aimed to explore the association between baseline serum TBA levels and T2DM status in ACS patients and to investigate the predictive power and value of TBA levels for a 2-year risk of MACEs in ACS patients following PCI.

## Materials and methods

### Study participants and procedures

It was a prospective cohort study conducted at a single center. Having screened 2132 consecutive patients diagnosed with CAD at First Affiliated Hospital of Xi’an Jiaotong University during January 2013 and February 2014, a total of 425 patients who were diagnosed with ACS, underwent PCI, and had fulfilled the follow-up were eventually recruited in this study. The detailed inclusion and exclusion criteria are shown in Supplementary Table 1 as previously described^11^. The flowchart is shown in Supplementary Fig. 1. Follow-up was conducted by well-trained physicians of our team at 1, 3, 6, 12, 18 and 24 months after PCI and once per year thereafter. Face-to-face follow-up was performed during routine outpatient clinic visits or coincided with hospital admissions. Telephone contacts were attempted if the participants could not come to the clinic or after discharge. The follow-up data were subsequently entered into case report form (CRF). The follow-up ended when the first MACEs occurred. All participants underwent follow-up until March 31, 2016. The median follow-up time was 27.8 months. Three-hundred and six (72%) of the patients had complete clinical face-to-face follow-up, while one-hundred and nineteen (28%) of the patients underwent telephone follow-up. Approval of the Ethics Committee of Xi’an Jiaotong University was obtained (Ethical approval number: XJTU1AF2012LSK-312). Patients signed the consent form to authorize follow-up through face-to-face or telephone interviews.

### Data collection

Baseline information included demographic characteristics (age, sex, etc.), known risk factors (smoking, family history, hypertension, etc.), clinical indices (systolic and diastolic blood pressure, ejection fraction) and baseline laboratory data (liver function, serum TBA, blood glucose, myocardial enzyme spectrum, and blood lipids, etc.). Data was collected directly from the electronic medical record into CRF and subsequently entered into the electronic database.

Composite endpoints, including all-cause death, cardiac death, unstable angina (UA), nonfatal MI, urgent coronary revascularization (included stent thrombosis and in-stent restenosis), heart failure, and cerebrovascular events (included TIA, ischemic stroke or cerebral bleeding), were defined as MACEs^12^ (Supplementary Table 1). UA was defined as clinical evidence including prolonged signs/symptoms of ischemia (< 30 min) or without changes in electrocardiographic ST-segment and elevated biomarkers for myocardial necrosis. MI was defined as elevated biomarkers for myocardial necrosis and clinical evidence including prolonged signs/symptoms of ischemia (> 30 min) or changes in electrocardiographic ST-segment. Stroke was defined as sudden onset of the loss of global or focal cerebral function persisting for more than 24 h. Heart failure was defined if the patients were in New York Heart Association (NYHA) functional class ≥ II, had a left ventricular ejection fraction ≤ 40% (heart failure with reduced ejection fraction), and had a N-terminal pro-B-type natriuretic peptide concentration ≥ 125 pg/mL^13^.

After admission, fasting peripheral blood samples were collected from patients before PCI. These laboratory tests were performed by experts from the Biochemistry Center of First Affiliated Hospital of Xi’an Jiaotong University using standard biochemical techniques. According to the manufacturer’s protocols, a reagent-based enzymatic cycling method (Pureauto S TBA, SEKISUI MEDICAL Co., Ltd.) was used to measure serum TBA concentrations on a Hitachi Chemistry Analyzer 7600. The normal maximum concentration of serum TBA was 10 µmol/L.

### Statistical analysis

Statistical analyses were performed using EmpowerStats (http://www.empowerstats.com/) and SPSS software (version 18.0; SPSS Inc., Chicago, IL, USA). Continuous variables were presented as the mean ± standard deviation (SD) if normally distributed or median interquartile range (IQR) otherwise. Categorical variables were expressed in terms of numbers of cases and frequencies. Differences in the parameters among groups were analyzed using analysis of variance (ANOVA) for normally distributed variables, the Kruskal–wallis analysis was used for nonnormally distributed continuous variables, and the chi-square test was used for categorical variables. An analysis of linear regression was performed to determine the correlation between the variables. The concentration of 3.1 µmol/L, which was the median TBA concentration for all participants, and 10 µmol/L, which was the normal maximum concentration of serum TBA, were used as a basis for grouping. We classified participants into three groups (low-level: ≤ 3.1 µmol/L; medium-level: 3.1–10.0 µmol/L; high-level > 10.0 µmol/L). Kaplan–Meier curves were employed to analyze the differences between groups in predicting MACEs over a 2-year follow-up period. To calculate MACEs risk, time-dependent receiver operating characteristic (ROC) and Cox proportional hazards regression models were employed. Multivariate multiple imputation was performed to impute missing values, using EmpowerStats. Statistics were considered significant at a value of *P* < 0.05.

### Ethics statement

Approval of the Ethics Committee of Xi’an Jiaotong University (Ethical approval number: XJTU1AF2012LSK-312) was obtained, and the written informed consents were obtained from all the participants. The study conformed to the Declaration of Helsinki and collected the required data from the clinical records without clinical intervention to protect patients' privacy.

## Results

### Basic characteristics and MACEs rates of participants

In total, 425 patients diagnosed with ACS who were treated with PCI and completed follow-up were enrolled in this research. The baseline characteristics of all patients, those with T2DM, and those without T2DM are shown in Table 1. No significant differences were observed in baseline demographics, clinical parameters, and laboratory data, except for age, history of smoking, alanine aminotransferase (ALT), aspartate aminotransferase (AST), creatine kinase isoenzymes (CK), and TBA concentrations (*P* < 0.05). At baseline, Serum TBA concentration in the total population was 3.1 (1.9–5.1) µmol/L. Serum TBA in diabetic patients was higher than non-diabetic patients (3.6 [1.9–5.4] vs. 3.0 [1.9–5.0] µmol/L), with a statistical difference. Table 2 shows the distribution of MACEs. Among the full cohort of 425 participants, MACEs occurred in 162 participants (38.1%). However, MACEs occurred in 37.6% in non-T2DM group versus 40.2% in T2DM group, resulting in no statistical difference in the risk of MACEs between the groups.

**Table 1:** Basic characteristics of ACS patients with T2DM, without T2DM and overall.

Variables	Overall (n = 425)	Non-T2DM (n = 338)	T2DM (n = 87)	*P* value
TBA, µmol/L, median (IQR)	3.1 (1.9–5.1)	3.0 (1.9–5.0)	3.6 (1.9–5.4)	**0.015**
Age, mean (SD)	60.5 (10.5)	59.6 (10.4)	63.8 (9.9)	** < 0.001**
Male sex, n, %	336, 79.1%	273, 80.8%	63, 72.4%	0.088
BMI, kg/m^2^, mean (SD)	25.0 (3.3)	25.1 (3.3)	24.9 (3.2)	0.712
Past MI, n, %	69, 16.2%	58, 17.2%	11, 12.6%	0.308
Past PCI or CABG, %	83, 19.5%	66, 19.5%	17, 19.5%	0.998
Smoking, n, %	242, 56.9%	203, 60.1%	39, 44.8%	**0.011**
Family history, n, %	170, 40.0%	136, 40.2%	34, 39.1%	0.844
Hypertension, n, %	224, 52.7%	173, 51.2%	51, 58.6%	0.215
Systolic pressure, mmHg, median (IQR)	120 (114–140)	120 (112–140)	126 (119–140)	0.214
Diastolic pressure, mmHg, median (IQR)	80 (70–84)	80 (70–84)	80 (70–84)	0.734
Ejection fraction, %, median (IQR)	62.0 (49.0–68.0)	62.0 (49.0–68.0)	60.0 (45.5–68.0)	0.333
TG, mmol/L, median (IQR)	1.4 (1.1–2.0)	1.4 (1.1–1.9)	1.4 (1.0–2.0)	0.667
LDL-C, mmol/L, mean (SD)	2.2 (0.8)	2.2 (0.8)	2.1 (0.8)	0.245
HDL-C, mmol/L, mean (SD)	0.9 (0.2)	0.9 (0.2)	0.9 (0.2)	0.250
ApoA, mmol/L, mean (SD)	1.1 (0.2)	1.1 (0.2)	1.1 (0.2)	0.754
ALT, U/L, median (IQR)	26.8 (17.3–45.4)	29.1 (17.8–48.9)	22.6 (16.4–38.3)	**0.017**
AST, U/L, median (IQR)	27.0 (18.9–54.7)	27.8 (19.6–62.4)	22.0 (17.4–35.9)	**0.002**
Blood glucose, mmol/L, median (IQR)	5.6 (4.9–6.9)	5.5 (4.9–6.8)	5.6 (5.0–7.1)	0.554
hsCRP, mg/dL, median (IQR)	1.4 (0.7–3.2)	1.3 (0.6–3.2)	1.4 (0.7–2.9)	0.935
CK, U/L, median (IQR)	107.7 (66.0–286.9)	117.1 (69.5–353.1)	84.0 (57.9–153.9)	**0.002**
CKMB, U/L, median (IQR)	15.6 (11.6–32.5)	15.9 (11.4–38.7)	14.7 (12.1–24.0)	0.257
pro-BNP, pg/mL, median (IQR)	267.8 (94.9–831.4)	264.9 (80.4–827.7)	289.3 (145.9–813.1)	0.213
Medication at discharge				
Aspirin, n, %	425, 100%	338, 100%	87, 100%	
Clopidogrel, n, %	425, 100%	338, 100%	87, 100%	
Statin, n, %	417, 98.1%	331, 97.9%	86, 98.9%	1.000
ACEI/ARB, n, %	385, 90.6%	309, 91.4%	76, 87.4%	0.247
β-blocker, n, %	374, 88.0%	298, 88.2%	76, 87.4%	0.836
ACS type				0.673
UA, n, %	239, 56.4%	189, 55.9%	50, 57.5%	
STEMI, n, %	126, 29.7%	103, 30.5%	23, 26.4%	
NSTEMI, n, %	60, 14.1%	46, 13.6%	14, 16.1%	
NYHA classification				0.163
I, n, %	154, 36.2%	131, 38.8%	23, 26.4%	
II, n, %	242, 56.9%	186, 55.0%	56, 64.4%	
III, n, %	20, 4.7%	15, 4.4%	5, 5.7%	
IV, n, %	9, 2.1%	6, 1.8%	3, 3.4%	

Significant values are in bold.

**Table 2:** MACEs rates of ACS patients with T2DM, without T2DM and overall.

End points	Overall (n = 425)	Non-T2DM (n = 338)	T2DM (n = 87)	*P* value
MACEs, n, %	162, 38.1%	127, 37.6%	35, 40.2%	0.649
UA and non-fatal MI, n, %	83, 19.5%	64, 18.9%	19, 21.8%	0.542
Urgent coronary revascularization, stent thrombosis and in-stent restenosis, n, %	20, 4.7%	13, 3.8%	7, 8.0%	0.099
Heart failure, n, %	38, 8.9%	30, 8.9%	8, 9.2%	0.926
Cerebrovascular events, n, %	20, 4.7%	12, 3.6%	8, 9.2%	**0.027**
Cardiac death, n, %	17, 4.0%	15, 4.4%	2, 2.3%	0.364
All-cause death, n, %	23, 5.4%	19, 5.6%	4, 4.6%	0.707

Significant values are in bold.

### Correlation between T2DM status and TBA in patients

Linear regression analysis was employed to investigate the relationship between the T2DM status in patients with ACS and serum TBA concentrations (Table 3). Subjects with T2DM had higher TBA levels than those without T2DM (β = 1.7, 95% CI 0.3–3.0). Multivariate-adjusted linear regression models were used. Of these, model 1 adjusted for age and sex, model 2 adjusted for age, sex, hypertension, family history, MI history, PCI/coronary artery bypass grafting (CABG) history, NYHA classification, ACS type, LDL-C, blood glucose, and hypersensitive C-reactive protein (hs-CRP). Compared to non-T2DM, those with T2DM had higher TBA levels. The adjusted *P*-values remained significant (Model 1: β = 1.6, 95% CI 0.3–3.0; Model 2: β = 2.3, 95% CI 0.3–4.3).

**Table 3:** Linear regression analysis between T2DM status and TBA levels. Model 1 adjusted for: Age, Sex. Model 2 adjusted for: Age, Sex, Hypertension, Family history, Past MI, Past PCI or CABG, NYHA classification, ACS type, LDL-C, hsCRP and Blood glucose.

Patients	Non-adjust	Model 1	Model 2
	β (95% CI), *P* value	β (95% CI), *P* value	β (95% CI), *P* value
Non-T2DM	0 (ref)	0 (ref)	0 (ref)
T2DM	1.7 (0.3, 3.0), **0.015**	1.6 (0.3, 3.0), **0.019**	2.3 (0.3, 4.3),** 0.025**

Significant values are in bold.

The patients were further grouped based on sex and age (participants ranged from 31 to 89 years). We then performed a stratified analysis according to sex and age (Supplementary Table 2). Compared to non-T2DM individuals, male individuals with T2DM showed higher TBA levels (β = 2.0, 95% CI 0.3–3.6). The adjusted β value remained significant (β = 2.9; 95% CI 0.3–5.4). Compared to the non-T2DM group, the female T2DM population showed no significant differences in TBA levels. The patients were grouped into three groups based on their age range (≤ 49 y, 50–69 y, and ≥ 70 y). Compared to non-T2DM subjects, subjects who were 50–69 years old had higher TBA levels (β = 2.5, 95% CI 0.6–4.4). In the multivariate-adjusted models (adjusted for hypertension, family history, MI history, PCI or CABG history, NYHA classification, ACS type, and LDL-C, hsCRP, and blood glucose levels), the adjusted *P*-values remained significant. However, no significant differences were observed in individuals under 50 years of age or over 70 years of age with T2DM compared to those without T2DM. The interaction analysis revealed a non-significant interaction effect across all subgroups.

### Time-dependent ROC curves of TBA as a marker to predict MACEs

**Figure 1:**
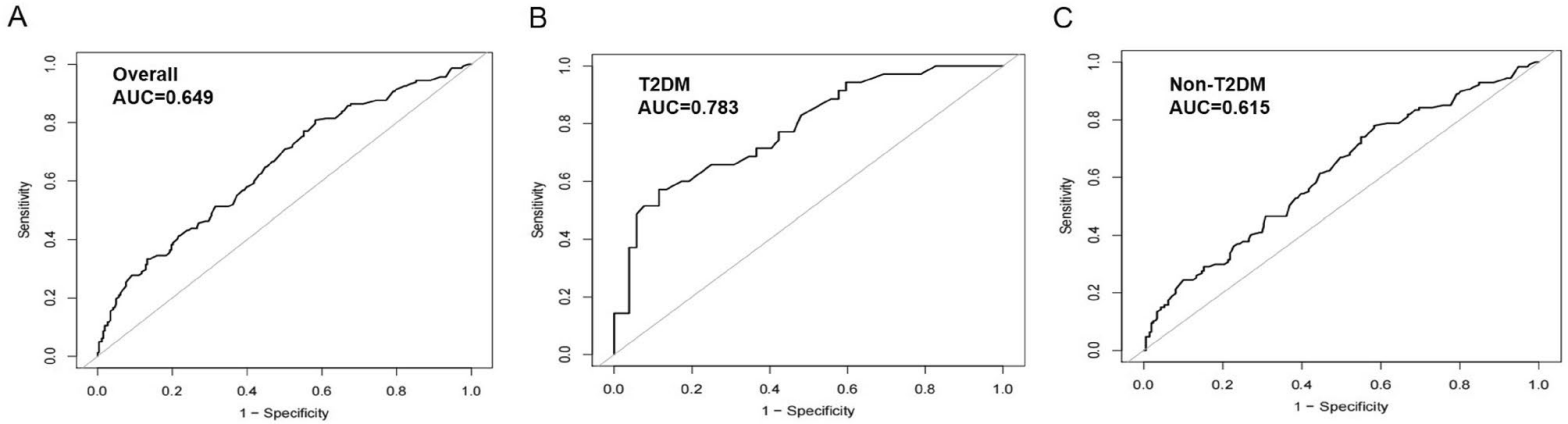
The ROC curves of TBA as a marker to predict MACEs in the whole patients (**A**), patients with T2DM (**B**), and without T2DM (**C**), following PCI (all *P* < 0.05). *ROC* Receiver operating characteristic; *TBA* Total bile acids; *MACEs* Major adverse cardiovascular events; *DM* Diabetes mellitus; *PCI* Percutaneous coronary intervention; *AUC* Area under ROC curves.

Figure 1 shows the time-dependent ROC curves of baseline serum TBA levels for predicting the occurrence of MACEs in patients with ACS following PCI. The area under the ROC curve (AUC) in the whole patient group, ACS-T2DM patient group and ACS-Non-T2DM patient group was 0.649 (95% CI 0.595–0.703), 0.783 (95% CI 0.685–0.881) and 0.615 (95% CI 0.553–0.677), respectively (Supplementary Table 3).

### Characteristics and MACEs rates of low-level, medium-level, and high-level TBA groups

Based on the median level and the normal upper limit of TBA, the three groups (low-level, high-level, and medium-level) presented an upward trend in TBA concentration. Supplementary Table 4 lists the characteristics and the MACEs rates in the three groups. No significant differences were observed except for ALT, AST, and the frequency of T2DM (*P* < 0.05). Patients in the medium-level TBA subgroup had a significantly lower MACEs incidence than those in the high-level TBA subgroup but a higher incidence of MACEs than those in the low-level TBA subgroup (*P* < 0.05). For individual MACEs component events, the incidence of UA and non-fatal MI increased steadily with the TBA level (*P* = 0.006); however, significant differences were not observed for other individual MACEs component events (Supplementary Table 4).

### Cox regression and Kaplan–Meier analysis of TBA as a marker to predict 2-year MACEs

Univariate and multivariate Cox regression analyses were carried out to determine the clinical predictive value of serum TBA concentration for MACEs over a median of 27.8 months of follow-up in ACS patients following PCI. Univariate Cox regression analysis revealed that age, hypertension, NYHA class II and III, and baseline TBA level were positively correlated with MACEs, whereas LDL-C, AST, and CK-MB levels were negatively correlated with MACEs (Table 4).

**Table 4:** Univariate and multivariate Cox regression analysis of MACEs.

Variables	Univariate analysis			Multivariate analysis		
	HR	95% CI	*P* value	HR	95% CI	*P* value
TBA	1.031	1.018, 1.045	** < 0.001**	1.031	1.014, 1.048	** < 0.001**
Age	1.025	1.009, 1.040	**0.001**	1.017	0.999, 1.035	0.066
Male sex	0.971	0.664, 1.421	0.881	0.938	0.625, 1.408	0.757
BMI	0.998	0.949, 1.049	0.936			
Smoking	1.081	0.789, 1.481	0.629			
Family history	0.881	0.643, 1.208	0.431			
Hypertension	1.390	1.017, 1.899	**0.039**	1.182	0.819, 1.706	0.372
T2DM	1.082	0.743, 1.575	0.682	0.899	0.600, 1.347	0.605
NYHA classification						
I	1			1		
II	1.594	1.126, 2.258	**0.009**	0.987	0.653, 1.494	0.952
III	3.510	1.966, 6.269	** < 0.001**	1.921	0.963, 3.831	0.064
IV	0.970	0.382, 2.464	0.949	0.831	0.310, 2.228	0.713
Systolic pressure	1.008	0.999, 1.015	0.057	1.000	0.987, 1.014	0.977
Diastolic pressure	1.011	0.998, 1.025	0.090	1.012	0.991, 1.034	0.270
Ejection fraction	0.992	0.980, 1.005	0.230			
Past MI	1.331	0.904, 1.961	0.148			
Past PCI or CABG	1.263	0.875, 1.825	0.214	1.119	0.749, 1.672	0.584
TG	0.943	0.801, 1.110	0.481			
LDL-C	0.767	0.630, 0.935	**0.009**	0.787	0.630, 0.984	**0.035**
HDL-C	0.562	0.261, 1.212	0.142			
ALT	0.992	0.985, 0.999	0.233	1.000	0.992, 1.007	0.926
AST	0.996	0.993, 0.999	**0.008**			
Blood glucose	0.983	0.910, 1.061	0.657			
hsCRP	0.966	0.889, 1.050	0.416			
CKMB	0.995	0.991, 0.998	**0.002**	0.996	0.992, 0.999	**0.036**
pro-BNP	1.000	0.999, 1.000	0.064	1.000	0.999, 1.000	0.091

Significant values are in bold.

We then calculated the variance inflation factor (VIF) scores, and the VIF scores of all variables were below 10. We selected confounders based on their associations with the outcomes of interest or a change-in-effect estimate of more than 10%. Multivariate analysis showed that TBA had a slight value for independently predicting 2-year MACEs risk in patients with ACS after PCI. Compared with the crude regression analysis, the association did not change markedly after adjusting for age, sex, T2DM, hypertension, systolic pressure, diastolic pressure, past PCI or CABG, NYHA classification, ALT, LDL-C, CKMB and pro-BNP in the multivariate regression analysis (Table 4).

We also analyzed the associations between serum TBA concentration and individual clinical events of MACEs using univariate Cox regression models. As displayed in Supplementary Table 5, baseline serum TBA concentration was independently predictive of two clinical events of MACEs (UA and non-fatal MI, and urgent coronary revascularization, stent thrombosis and in-stent restenosis), over a median of 27.8 months of follow-up in ACS patients following PCI.

Next, we explored the values of TBA for predicting MACEs in subgroups with different TBA levels. The results of univariate Cox regression analysis showed that the TBA levels were associated with increased MACEs in low-level group and medium-level group, both in the whole population and ACS-T2DM patients (Table 5). Additionally, we note that for every 1.0 µmol/L increase in TBA, the increased risk of MACEs in diabetics was higher than that in whole participants when their baseline TBA concentration was greater than 3.1 µmol/L but less than 10.0 µmol/L (HR: 2.961 vs. 1.542). When TBA was lower than 3.1 µmol/L, for every 1.0 µmol/L increase in TBA, the risk of MACEs increased by 20.1%, 43.9% and 16.0% in the overall, non-diabetes, and diabetes cohorts, respectively. While when TBA > 10.0 µmol/L, there was no association between TBA and the risk of MACEs in all the cohorts above in Cox regression models (Table 5).

**Table 5:** Cox regression subgroup analysis of TBA for predicting MACEs in ACS patients with DM, without DM and whole.

Cohorts	HR	95% CI	*P* value
Whole			
TBA ≤ 3.1 µmol/L	1.206	1.067, 1.364	**0.003**
TBA: 3.1–10.0 µmol/L	1.542	1.086, 2.188	**0.015**
TBA > 10.0 µmol/L	1.020	0.994, 1.048	0.134
ACS-T2DM			
TBA ≤ 3.1 µmol/L	1.439	1.123, 1.845	**0.004**
TBA: 3.1–10.0 µmol/L	2.961	1.085, 8.083	**0.034**
TBA > 10.0 µmol/L	1.019	0.989, 1.050	0.209
ACS-non-T2DM			
TBA ≤ 3.1 µmol/L	1.160	1.004, 1.339	**0.044**
TBA: 3.1–10.0 µmol/L	1.366	0.937, 1.991	0.105
TBA > 10.0 µmol/L	1.017	0.823, 1.257	0.878

Significant values are in bold.

**Figure 2:**
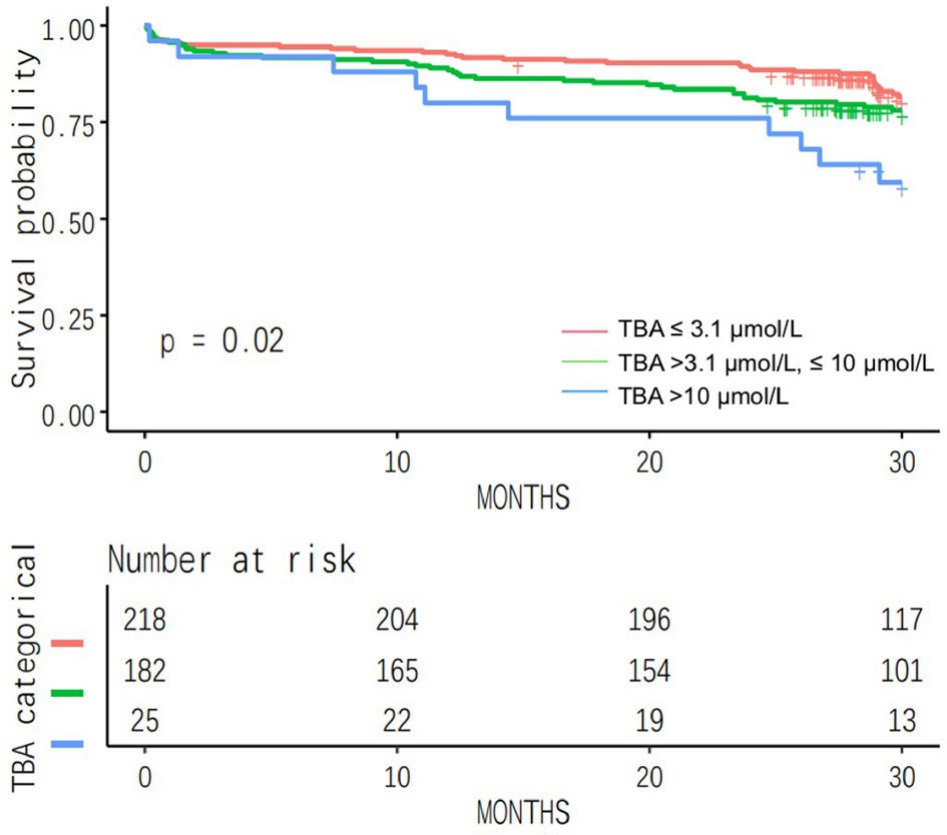
Kaplan–Meier survival curves. The prediction of MACEs over a 2-year follow-up in low-level (≤ 3.1 µmol/L), medium-level (> 3.1 µmol/L, ≤ 10 µmol/L) and high-level (> 10 µmol/L) TBA groups in ACS patients after PCI.

Kaplan–Meier curves were employed to analyze the differences between subgroups with different TBA levels in predicting MACEs. Slightly differed from the univariate Cox analysis, the Kaplan–Meier survival analysis illustrated that the incidence of MACEs within 30 months after PCI was higher among ACS patients with TBA levels in the highest group (Fig. 2).

## Discussion

We conducted this study, which included 425 patients with ACS after PCI, and conducted follow-up examinations (the median follow-up time was 27.8 months) to explore the clinical predictive values of baseline serum TBA concentration for the occurrence of MACEs. We found that serum TBA levels were higher in ACS patients who were diagnosed with T2DM than in those without T2DM. T2DM status in patients with ACS was related to baseline serum TBA concentration, especially in male and 50–69-year-old populations. Furthermore, the results of the current study indicated that the baseline serum TBA level was an independent predictor of MACEs in ACS patients who treated with PCI over a 2-year follow-up, especially in those diagnosed with T2DM, whose baseline TBA concentration were lower than 10.0 µmol/L. To the best of our knowledge, this is the first study to uncover the relationship between serum TBA levels and 2-year MACEs in patients with ACS after PCI in China.

BA is the final product of cholesterol metabolism. Most bile salts are reabsorbed into the distal ileum following secretion into the duodenum, while a small percentage of bile salts that escape resorption are converted by bacterial flora into secondary bile acids, which may be absorbed in the absence of passive processes, or excreted into feces^1^. In individuals without biliary tract obstruction or hepatocyte damage, the major components of serum TBA are reabsorbed BA following systemic circulation spillover. This process is known as enterohepatic circulation^14^. BA plays a vital role in cholesterol homeostasis. In this study, we found that T2DM status in patients with ACS was positively associated with baseline serum TBA concentrations, and this relationship persisted after multivariate analysis. In other words, patients with ACS and diabetes had higher levels of TBA (approximately 1.7 times) than those without diabetes. Recent studies have demonstrated that BA is involved in glucose and lipid metabolism, and that abnormal BA metabolism is closely linked to metabolic diseases^9,15^. The results of our study are in accordance with those of most existing studies. For example, when compared to non-T2DM controls, TBA levels in patients with T2DM significantly increased during the feeding state. The levels of TBA positively correlated with body mass index*,* blood pressure, TG, and insulin resistance (IR) index in patients with T2DM^16,17^. Changes in the composition of BA in the plasma have been observed in several clinical trials. Increased deoxycholic acid (DCA) and decreased chenodeoxycholic acid (CDCA) levels were observed in patients with T2DM^7,18^. Another study indicated that glyco-BA was elevated in T2DM patients when compared to non-T2DM controls^19^. As a result of these findings, TBA levels seem to increase in diabetic patients. In addition to lipid transport and intestinal absorption, BA facilitates the release of pro-inflammatory molecules and acts as an essential regulator of cell signaling pathways that regulate energy metabolism and modulate blood glucose and lipid homeostasis. BA metabolism may involve the precise feedback mechanisms of two receptors: farnesoid X receptor (FXR) and transmembrane G protein-coupled receptor 5 (TGR5)^18^. BA can activate FXR and TGR5, which improves glucose tolerance, insulin sensitivity, and energy metabolism^20,21^. In summary, TBA is an important risk factor for diabetes progression. Although the exact mechanism is not fully understood and the repeatability and authenticity of previous studies need to be further refined and verified, we speculate that elevated TBA levels might be either a manifestation or a leading cause of diabetic progression.

Sex differences in serum TBA levels remain controversial. Studies by Kawasaki et al. ^22^, Luo et al.^23^ and Li et al. indicated no statistical differences in circulating TBA between healthy individuals and patients with CAD, whereas Xie et al.^24^ found that compared to healthy females, males with healthy bodies had significantly higher circulating TBA. Here, we report that male patients with T2DM are approximately two times more likely to have high TBA levels than those without T2DM. However, when compared with patients without T2DM, female patients with T2DM showed no significant differences in TBA levels. One possible explanation for this is that the average bile acid pool in females is lower by a considerable amount than that in males. Further research is needed to confirm sex differences in blood TBA levels. In contrast, we found that patients with T2DM who were 50–69 years old had TBA levels approximately 2.5 times higher than those without T2DM. The differences remained significant after adjustments. Taken together, these findings suggest that male ACS patients with T2DM, who were 50–69 years old, were more inclined to have higher serum TBA levels, which may be instructive for clinicians and help them identify high-risk patients, optimize clinical decisions, and design individualized treatment plans for these patients.

CAD is usually classified into stable coronary heart disease or ACS. ACS, which includes UA, NSTEMI, and STEMI, is a common and severe CAD that leads to global morbidity and mortality^25^. Although revascularization and antithrombotic strategies have successfully reduced mortality in patients with ACS, the results are unsatisfactory, especially in patients diagnosed with T2DM^26^. Although TBA plays an important role in cholesterol homeostasis and diabetes progression, the relationship between the TBA levels and CAD remains controversial. Steiner et al.^27^ reported no differences in the serum TBA levels between patients with CAD and healthy subjects. Another study indicated that elevated serum TBA level was an important and independent predictor of coronary plaque instability based on coronary computed tomography angiography^28^. Some studies, however, have contradictory reports. Study from Wenyuan Li and co-workers^10^ revealed that patients diagnosed with CAD had lower TBA concentrations than those without CAD. A previously published article reported that circulating TBA levels were inversely related to 3-month mortality in patients with acute ischemic stroke^29^. Another research^30^ suggested that the lower the TBA levels in menopausal female patients with T2DM, the higher the occurrence rate of CAD. However, very few research have investigated the prediction of outcomes in patients with ACS and T2DM, especially after PCI. Our main results showed that the serum TBA level was an independent predictor of the 2-year risk of MACEs in patients with ACS after PCI. Notably, when baseline concentration of serum TBA was lower than 10.0 µmol/L, as the TBA concentration increased, the HRs for MACEs increased; Kaplan–Meier survival analysis indicated that a higher level of TBA had a greater predictive value as well. Although log-rank test and Cox proportional risk model have different assumptions and statistical properties, which may lead to inconsistent results in some cases, it is clear that the two methods gave consistent results. The underlying causes of the diverse findings are likely complicated. We can only speculate about the reasons for this discrepancy. Firstly, the race and the disease characteristics of the study population were different among studies. Multiple factors, including sex, age, smoking, obesity, and type of vascular lesions, contribute to the progression of MACEs after ACS, increasing the MACEs occurrence rate even after PCI^31^. We speculated that our study population have a higher incidence of MACE events and worse long-term prognosis than other patients. Secondly, we speculated that the potential protective effect of TBA illustrated by other studies may be related to its involvement as a signaling molecule in regulating cholesterol metabolic pathways, as well as its antiapoptotic effect. Alternatively, we note that the MACEs occurred in 38% of ACS patients during the follow-up period of 27.8 months. The MACEs rate in the current study was higher than that previously reported. For example, 24% from Zhong-Fei Lu et al., Beijing, China^32^, and 10% from Yun Wang et al., Hong Kong, China^33^. The diversity of MACEs rates among studies may arise due to the heterogeneity of study population and different treatment strategies and the definition of MACEs. In the present study, we included all-cause death, cardiac death, UA, non-fatal MI, urgent coronary revascularization, heart failure and cerebrovascular events as part of the definition of MACEs. UA in our study occurred more commonly in ACS patient following PCI. We proposed that high TBA levels were associated with the severity of coronary artery stenosis and risk coronary artery plaque. Additionally, ACS patients are often complicated with multivessel coronary disease (MVCD). They have more risk factors, may undergo more revascularization intervention and bypass surgery, have a higher incidence of MACEs than other patients with single vessel coronary disease or non-MVCD^34^.

Compared to patients without T2DM, those with T2DM usually display accelerated atherosclerosis progression and more serious clinical outcomes^35^. A wide range of adverse outcomes are associated with T2DM, including myocardial infarction, stroke, heart failure, atrial fibrillation, nephropathy, and mortality^36^. A large-scale clinical study revealed that among patients with T2DM and obesity, metabolic surgery had a close relationship with a lower risk of MACEs than nonsurgical management^37^. However, in our present study, univariate Cox proportional hazard model revealed no relation between T2DM and MACEs over a 2-year follow-up in ACS patients after PCI. The exact mechanisms responsible for the independent predictive factors for 2-year MACEs of TBA are not well defined. Although TBA is used as a CAD marker and closely relates to metabolism and inflammation, whether it can affect T2DM and thereby further influence the progression and prognosis of CAD is unclear. We speculate that, in the diabetic state, TBA in plasma might act on target receptors through blood circulation and participate in inflammatory responses and glucose metabolism by activating specific pathways at the level of molecules and signaling. The activation of BA-mediated signaling pathways may be related to enhanced inflammation thereby further influencing the progression and prognosis of CAD. Elevated TBA levels are the reflection of higher inflammation and disorder in metabolism in diabetic patients, who are at a higher risk of recurrent atherosclerotic cardiovascular disease. This hypothesis might be supported by the result of the current study that, when compared to non-T2DM patients, patients with T2DM had a higher risk of MACEs incidence over a median follow-up of 27.8 months, even after adjusting for confounding factors.

### Limitation

(1) The current study was a single-center study with a small sample size limited to patients with ACS following PCI in China. Our results were obtained in a homogeneous population of ACS patients and cannot be extrapolated to other population. A larger cohort from a multi-center study and different populations are needed to further refine our findings. (2) Only 87 patients with ACS and T2DM were enrolled in this investigation, which may have resulted in TBA presenting a non-normal distribution, leading to a small number of subjects in certain groups (high-level TBA group), which might have affected the reliability of the results. (3) MACEs were assessed by only two of the investigators in this study. In addition, the use of UA as a MACE event was also prone to bias. However, the diagnosis of UA was not only made according to symptoms. The patients with UA in this study all received coronary angiography and received coronary interventions if the in-charge cardiologist thought it would be beneficial for the patients. (4) There are still some possible factors which might have impacts on the outcomes, such as coronary lesion characteristics, have not been adjusted. (5) Only serum TBA concentrations were measured. Further investigation of the specific components of bile acids, such as ursodeoxycholic acid (UDCA), CDCA, and DCA, is required to validate our findings.

## Conclusions

Patients with ACS and T2DM had higher serum TBA levels. Elevated baseline serum TBA concentrations in patients with ACS following PCI were associated with a 2-year risk of MACEs, most likely attributable to T2DM status, suggesting that serum TBA levels may be an available, simple, and cost-effective biomarker with predictive value. These findings may help clinicians identify high-risk patients, optimize clinical decisions, and make individualized treatment designs, especially for patients with ACS and T2DM.

### Supplementary Information

Supplementary Figure 1.

Supplementary Information.

## Data Availability

Data used to support the findings of this study are included within the article, and the original records of the enrolled patients are available from the corresponding authors upon reasonable request.
