# Supplementary figures and images for: Predictive value of serum TBA for 2-year MACEs in ACS patients undergoing PCI: a prospective cohort study
Source: Sci Rep. 2024 Jan 19;14:1733. doi: 10.1038/s41598-023-50304-z (PMC10799034; doi:10.1038/s41598-023-50304-z)

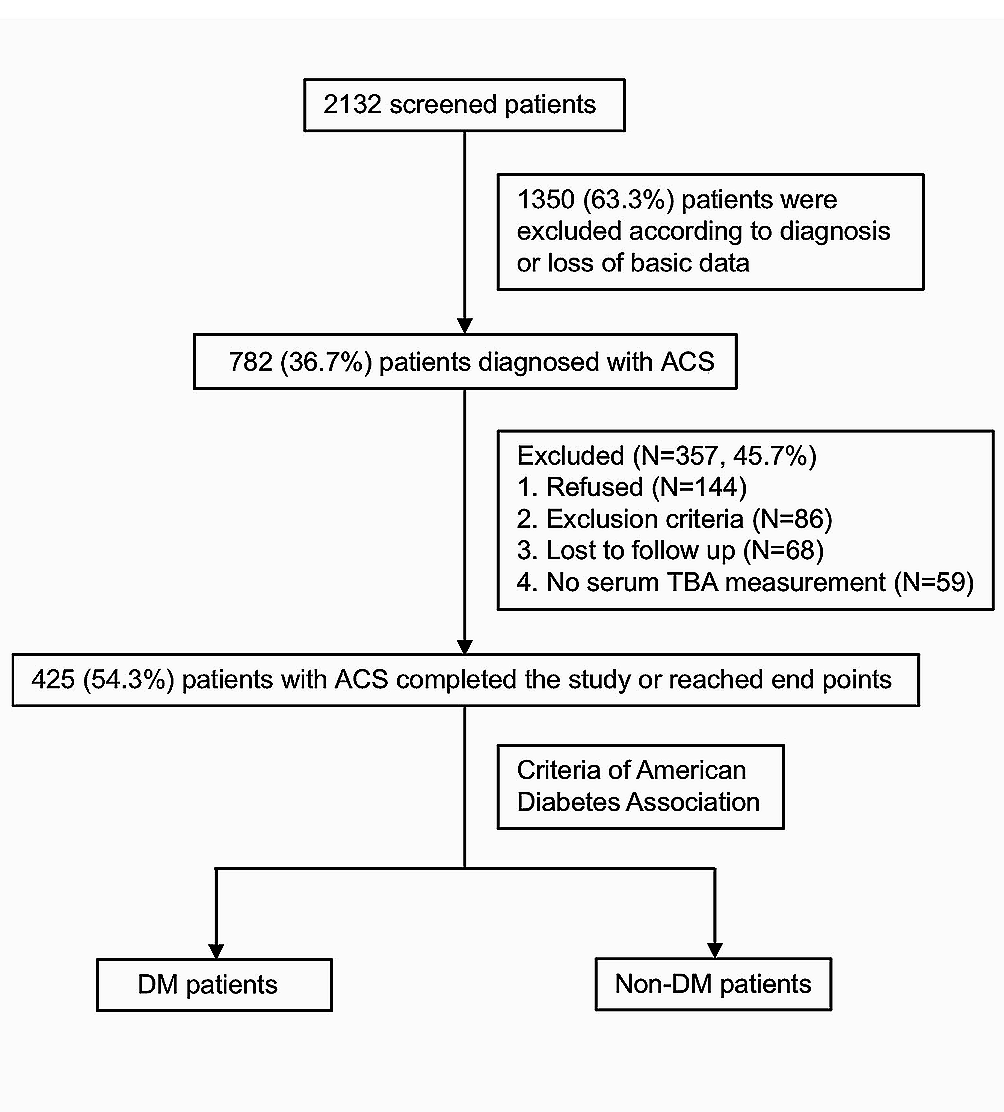

Supplement: Supplementary file 1 — Supplementary Figure 1. [file 41598_2023_50304_MOESM1_ESM.tiff]
