# Supplementary material for: Predictive value of serum TBA for 2-year MACEs in ACS patients undergoing PCI: a prospective cohort study
Source: Sci Rep. 2024 Jan 19;14:1733. doi: 10.1038/s41598-023-50304-z (PMC10799034; doi:10.1038/s41598-023-50304-z)
Supplement: Supplementary file 2 — Supplementary Information. [file 41598_2023_50304_MOESM2_ESM.docx]

**Predictive Value of Serum TBA for 2-Year MACEs in ACS Patients Undergoing PCI - A Prospective Cohort Study**

**Supplemental Materials**

**Supplementary Table 1 Inclusion, exclusion criteria and MACEs**

| Inclusion criteria | Consecutive patients who were diagnosed with ACS and underwent PCI in the First Affiliated Hospital of Medical College of Xi’an Jiaotong University between January 2013 and February 2014. The diagnosis of ACS included UA, NSTEMI and STEMI. (ACS and T2DM were defined based on the criteria of American Cardiology College and the American Diabetes Association) |
| --- | --- |
| Exclusion criteria | 1. Patients with severe renal dysfunction (defined using MDRD equation as eGFR <30mL/min) or liver dysfunction (serum ALT > 3 times the upper normal limit) 2. Patients with obstructive diseases of the biliary tract 3. Patients with severe infection and immune system diseases 4. Patients with malignant tumors and severe coagulation disorder or thrombocytopenia (platelets levels <100×10^9^/L) 5. Patients with severe cerebrovascular diseases 6. Patients who were pregnancy 7. Patients who were alcohol abuse (estimated >30 g/day) |
| Composite end points:  MACEs | 1. All-cause death and cardiac death 2. UA and non-fatal MI 3. Urgent coronary revascularization, stent thrombosis, in-stent restenosis 4. Heart failure 5. Cerebrovascular events (including TIA, cerebral bleeding or ischemic stroke) |

**Supplementary Table 2 Linear regression analysis between T2DM status and TBA levels in the pre-specified subgroups. Adjust model adjusted for: Hypertension, Family history, Past MI, Past PCI or CABG, NYHA classification, ACS type, LDL-C, hsCRP and Blood glucose.**

| Subgroups | Non-adjusted model | Adjust model |  |
| --- | --- | --- | --- |
|  | β (95% CI), *P* value | β (95% CI), *P* value |  |
| Sex: Males |  |  |  |
| Non-T2DM | 0 (ref) | 0 (ref) |  |
| T2DM | **2.0 (0.3, 3.6), 0.020** | **2.9 (0.3, 5.4), 0.029** |  |
| Females |  |  |  |
| Non-T2DM | 0 (ref) | 0 (ref) |  |
| T2DM | 0.6 (-1.4, 2.6), 0.533 | 0.5 (-2.3, 3.3), 0.721 |  |
| Age: ≤49y |  |  |  |
| Non-T2DM | 0 (ref) | 0 (ref) |  |
| T2DM | -1.1 (-3.3, 1.1), 0.331 | -1.6 (-5.9, 2.7), 0.472 |  |
| 50-69y |  |  |  |
| Non-T2DM | 0 (ref) | 0 (ref) |  |
| T2DM | **2.5 (0.6, 4.4), 0.012** | **3.1 (0.2, 6.0), 0.041** |  |
| ≥ 70y |  |  |  |
| Non-T2DM | 0 (ref) | 0 (ref) |  |
| T2DM | 0.2 (-1.5, 1.9), 0.801 | 1.1 (-1.7, 3.9), 0.463 |  |

**Supplementary Table 3 AUCs of baseline serum TBA levels in predicting the occurrence of MACEs in patients**

|  | AUC | 95% CI | *P* value |
| --- | --- | --- | --- |
| Overall | 0.649 | 0.595, 0.703 | <0.05 |
| T2DM | 0.783 | 0.685, 0.881 | <0.05 |
| Non-T2DM | 0.615 | 0.553, 0.677 | <0.05 |

**Supplementary Table 4 Basic characteristics and MACEs rates of patients in****low-level, medium-level and high-level** **TBA groups**

| Variables | TBA |  |  | *P* value |
| --- | --- | --- | --- | --- |
|  | low-level  (≤ 3.1 µmol/L) | medium-level  (3.1 - 10.0 µmol/L) | high-level  (> 10.0 µmol/L) |  |
|  | n=218 | n=182 | n=25 |  |
| TBA, µmol/L, median (IQR) | 2.0 (1.4-2.6) | 4.6 (3.8-6.5) | 14.2 (13.2-17.0) | **<0.001** |
| Age, mean (SD) | 60.0 (10.6) | 60.9 (10.4) | 61.2 (9.9) | 0.688 |
| Male sex, n, % | 176, 80.7% | 144, 79.1% | 16, 64.0% | 0.150 |
| BMI, kg/m^2^, mean (SD) | 24.8 (3.3) | 25.3 (3.1) | 24.6 (4.5) | 0.348 |
| Past MI, n, % | 35, 16.1% | 33, 18.1% | 1, 4.0% | 0.198 |
| Past PCI or CABG, % | 42, 19.3% | 38, 20.9% | 3, 12.0% | 0.571 |
| Smoking, n, % | 122, 56.0% | 107, 58.8% | 13, 52.0% | 0.745 |
| Family history, n, % | 83, 38.1% | 80, 44.0% | 7, 28.0% | 0.220 |
| Hypertension, n, % | 112, 51.4% | 101, 55.5% | 11, 44.0% | 0.476 |
| Type 2 DM, n, % | 39, 17.9% | 40, 22.0% | 8, 32.0% | **0.020** |
| Systolic pressure, mmHg, median (IQR) | 121 (116-140) | 120 (115-140) | 120 (110-130) | 0.816 |
| Diastolic pressure, mmHg, median (IQR) | 80 (70-84) | 80 (70-82) | 80 (70-85) | 0.836 |
| Ejection fraction, %, median (IQR) | 61.0 (48.0-68.0) | 62.0 (50.8-68.2) | 60.0 (52.0-69.0) | 0.520 |
| TG, mmol/L, median (IQR) | 1.4 (1.0-1.9) | 1.5 (1.1-1.9) | 1.5 (1.1-2.4) | 0.498 |
| LDL-C, mmol/L, mean (SD) | 2.2 (0.8) | 2.3 (0.8) | 2.1 (0.6) | 0.643 |
| HDL-C, mmol/L, mean (SD) | 0.9 (0.2) | 0.9 (0.2) | 0.9 (0.2) | 0.920 |
| ApoA, mmol/L, mean (SD) | 1.1 (0.2) | 1.1 (0.2) | 1.1 (0.2) | 0.644 |
| ALT, U/L，median (IQR) | 29.6 (18.3-50.6) | 23.9 (16.5-39.4) | 29.6 (18.2-39.3) | **0.035** |
| AST, U/L，median (IQR) | 29.1 (20.6-74.1) | 23.0 (17.8-44.8) | 26.9 (20.5-37.9) | **0.003** |
| Blood glucose, mmol/L，median (IQR) | 5.5 (5.0-6.9) | 5.7 (5.0-7.0) | 5.2 (4.6-5.7) | 0.069 |
| hsCRP, mg/dL, median (IQR) | 1.4 (0.7-3.2) | 1.4 (0.7-3.4) | 1.5 (0.5-2.7) | 0.473 |
| CK, U/L, median (IQR) | 116.3 (65.6-391.9) | 107.2 (66.2-213.9) | 91.6 (75.4-150.4) | 0.628 |
| CKMB, U/L, median (IQR) | 16.5 (11.7-43.1) | 15.0 (11.3-25.8) | 14.7 (11.5-17.9) | 0.175 |
| pro-BNP, pg/mL, median (IQR) | 288.4 (88.1-824.6) | 263.1 (103.5-850.3) | 240.6 (127.0-678.6) | 0.963 |
| Medication at discharge |  |  |  |  |
| Aspirin, n, % | 218, 100% | 182,100% | 25, 100% |  |
| Clopidogrel, n, % | 218, 100% | 182,100% | 25, 100% |  |
| Statin, n, % | 212, 97.2% | 180, 98.9% | 25, 100% | 0.571 |
| ACEI/ARB, n, % | 197, 90.4% | 163, 89.6% | 25, 100% | 0.242 |
| β-blocker, n, % | 197, 90.4% | 155, 85.2% | 22, 88.0% | 0.281 |
| ACS type |  |  |  | 0.272 |
| UA, n, % | 116, 53.2% | 107, 58.8% | 16, 64.0% |  |
| STEMI, n, % | 72, 33.0% | 46, 25.3% | 8, 32.0% |  |
| NSTEMI, n, % | 30, 13.8% | 29, 15.9% | 1, 4.0% |  |
| NYHA classification |  |  |  | 0.294 |
| I, n, % | 90, 41.3% | 54, 29.7% | 10, 40.0% |  |
| II, n, % | 114, 52.3% | 115, 63.2% | 13, 52.0% |  |
| III, n, % | 9, 4.1% | 9, 4.9% | 2, 8.0% |  |
| IV, n, % | 5, 2.3% | 4, 2.2% | 0, 0.0% |  |
| MACEs, n, % | 65, 29.8% | 79, 43.4% | 18, 72.0% | **<0.001** |
| UA and non-fatal MI, n, % | 30, 13.8% | 45, 24.7% | 8, 32.0% | **0.006** |
| Urgent coronary revascularization, stent thrombosis, and in-stent restenosis, n, % | 5, 2.3% | 13, 7.1% | 2, 8.0% | 0.054 |
| Heart failure, n, % | 15, 6.9% | 19, 10.4% | 4, 16.0% | 0.205 |
| Cerebrovascular events, n, % | 6, 2.8% | 12, 6.6% | 2, 8.0% | 0.142 |
| Cardiac death, n, % | 12, 5.5% | 3, 1.6% | 2, 8.0% | 0.084 |
| All-cause death, n, % | 14, 6.4% | 6, 3.3% | 3, 12.0% | 0.126 |

**Supplementary Table 5** **Univariate Cox regression analysis between** **serum TBA concentration and individual clinical events of MACEs.**

| Clinical events | HR | 95% CI | *P* value |
| --- | --- | --- | --- |
| UA and non-fatal MI | 1.055 | 1.033, 1.076 | **<0.001** |
| Urgent coronary revascularization,  stent thrombosis and in-stent restenosis | 1.037 | 1.015, 1.060 | **<0.001** |
| Heart failure | 1.026 | 0.995, 1.057 | 0.100 |
| Cerebrovascular events | 1.017 | 0.968, 1.070 | 0.504 |
| Cardiac death | 1.013 | 0.957, 1.072 | 0.657 |
| All-cause death | 1.025 | 0.993, 1.057 | 0.129 |

**Supplementary Figure Legends**

**Supplementary Figure 1** Flow chart. ACS: acute coronary syndrome; TBA: total bile acids.
